# Dentistry and the Gold Excise

**Published:** 1920-08

**Authors:** 


### DENTISTRY AND THE GOLD EXCISE

Under the title, "Our Vanishing Gold Reserve," the American Mining Congress, Washington, D. C., has issued a pamphlet embodying data covering the production and consumption of gold in the United States which clearly justify the disturbing conclusion stated in its title. It is evident that our gold reserve under present conditions is vanishing and that it will vanish completely unless controlling measures are adopted and enforced.

The figures indicate that the gold production of the United States has declined from one hundred and one millions in 1915 to fifty-eight millions in 1919, a loss of forty-two per cent. in four years. Decrease in gold production has reached a point where the total value of gold mined in the United States is less than the annual consumption of the metal in the arts, a condition that seriously threatens the status of our monetary reserve in gold —the basis of our currency system.

As an attempt to practically remedy this threatening state of affairs, a bill (H. R. 13201) was introduced by Hon. Louis T. McFadden on March 22nd, "To protect the gold reserves of the United States from industrial depletion,'' by imposing an excise tax of $0.50 a pennyweight ($10 per ounce), to be collected on the sale of all articles containing gold or gold used for other than monetary purposes, thereby creating a fund from which the gold producer is to receive $10 for every newly-produced ounce. Since this transaction is confined to the production and sale of gold as a commodity only, and without reference to its monetary use, it can not in any way influence the monetary status of the metal. The propriety of the principle involved in this proposed legislation can not, we think, be seriously questioned so far as its equity is concerned, when we clearly realize the distinction between the monetary function of gold and its status as a commodity or basic material for certain arts and manufactures. As the world's standard of monetary value the unit value of gold must be stabilized at a fixed unit standard by common consent, as a basis for all commercial value and transactions. On the other hand, as the basic material for arts and manufactures into which the metal enters, it becomes a commodity directly influenced in value by the law of supply and demand.

The circumstances arising out of the world war which have profoundly influenced the factors of supply and demand and created a new scale of values for all commodities and the necessities of life in general have had their direct effect in sending the value of gold as a commodity skyward in company with all other things.

Taking the wholesale index price number of all commodities in 1914 as 100, the index price number of these same commodities in 1919 is 212. So that had the gold producer shared in this increase of value he would have received 112 per cent. advance for his output of gold beyond what he actually did receive at the bullion value. Or specifically in addition to the $58,500,000, the production for 1919, he would have received $65,500,000.

As a matter of fact, the proposed excise of $10 per ounce on manufactured gold is offered as a means whereby the consumer of commodity gold shall pay a fair share of its actual market value and thus relieve the Government of the burden which it has carried in maintaining gold as a commodity at its monetary value and providing means by which the producer of gold should be assured of a fair margin of profit as the stimulus to increased production. Mr. McFadden's bill since its introduction has been amended as follows:

"That on account of the impracticability of suitably stamping finished dental restorative appliances, the Internal Revenue Department shall further prescribe such rules and regulations for the collection of the tax provided herein upon all gold used for dental purposes as will equitably protect the interest of the public; that all gold used by the Government for dental, medical and surgical purposes, and all gold employed in dental services rendered to war-risk insurance patients by the United States Public Health Service, shall be exempt from the excise provided herein; and that all gold used in corrective and restorative dental work for children of both sexes not over the age of fifteen, and all gold used in dental infirmaries conducted for the benefit of the poor and not for private profit, shall be exempt from the excise provided herein."

The foregoing provision for the exemption of the several cases specified is clearly in recognition of the policy of exempting from excise tax all humanitarian activities involving life and health, education, religion, etc., a principle practically recognized in all phases of our national, state and civic legislation. It is therefore eminently proper that the exemptions provided in the above quoted amendment should become part of the proposed law. But why recognize the principle involved and then arbitrarily limit its practical application? It has been the misfortune of dentistry simply because it makes use of gold and other precious metals in its service to be listed among the arts and crafts in various governmental classifications related to taxation; overlooking the fundamental fact that dentistry is a recognized and fully established department of the science and art of healing. This fact in its broad lines is already recognized by the Government of the United States in the official status which is accorded to dentistry in connection with the army and navy, and more recently in connection with the public health service.

The use to which gold is put by the dental profession is essentially a therapeutic use for the prevention and cure of disease, the relief of distress, the restoration of function and all material used for such purposes which mean ultimately the conservation of the public health, should be, indeed are in principle, exempt from taxation. Not over one-fourth of the gold classified as gold used in the arts is used in dentistry, medicine and surgery and by opticians. The balance is used for adornment or for luxurious articles that go to a clientélé amply able to pay for them at their market value. These should bear the tax, and the fraction that goes to the relief of human suffering should be exempt.

We submit that the dental profession of America should unite in emphasizing that point of view in connection with the pending action of the Congress on the McFadden bill by asking Congress to specifically exempt from the excise all gold used in dentistry, medicine and surgery.

If the criticism be made that such an attitude is selfish or unpatriotic, as has been already intimated, the answer is recorded in the reaction of the dental profession as a whole to the demands made upon it by the World War. On that we can rest our case.—Editorial in *Dental Cosmos,* for May, 1920.

